# Fibroblast growth factor 21 in heart failure

**DOI:** 10.1007/s10741-022-10268-0

**Published:** 2022-08-27

**Authors:** William Tucker, Bradley Tucker, Kerry-Anne Rye, Kwok Leung Ong

**Affiliations:** 1 Lipid Research Group, School of Medical Sciences, University of New South Wales, Sydney, NSW Australia; 2 Rural Clinical School, Faculty of Medicine, University of New South Wales, Sydney, NSW Australia; 3 Royal Prince Alfred Hospital, Camperdown, NSW Australia

**Keywords:** Biomarker, Cardiomyocyte, Fibroblast growth factor 21, Heart failure

## Abstract

Fibroblast growth factor 21 (FGF21) is a peptide hormone involved in energy homeostasis that protects against the development of obesity and diabetes in animal models. Its level is elevated in atherosclerotic cardiovascular diseases (CVD) in humans. However, little is known about the role of FGF21 in heart failure (HF). HF is a major global health problem with a prevalence that is predicted to rise, especially in ageing populations. Despite improved therapies, mortality due to HF remains high, and given its insidious onset, prediction of its development is challenging for physicians. The emergence of cardiac biomarkers to improve prediction, diagnosis, and prognosis of HF has received much attention over the past decade. Recent studies have suggested FGF21 is a promising biomarker candidate for HF. Preclinical research has shown that FGF21 is involved in the pathophysiology of HF through the prevention of oxidative stress, cardiac hypertrophy, and inflammation in cardiomyocytes. However, in the available clinical literature, FGF21 levels appear to be paradoxically raised in HF, potentially implying a FGF21 resistant state as occurs in obesity. Several potential confounding variables complicate the verdict on whether FGF21 is of clinical value as a biomarker. Further research is thus needed to evaluate whether FGF21 has a causal role in HF, and whether circulating FGF21 can be used as a biomarker to improve the prediction, diagnosis, and prognosis of HF. This review draws from preclinical and clinical studies to explore the role of FGF21 in HF.

## Introduction

Heart failure (HF) is a chronic, progressive condition characterised by an impairment in ventricular filling and/or a reduction of the cardiac ejection fraction due to structural and/or functional defects in the myocardium [1, 2]. It is not denoted by a single pathological diagnosis but rather a clinical syndrome with marked symptoms such as dyspnoea and fatigue together with associated signs, namely elevated jugular venous pressure, pulmonary crackles, and peripheral oedema [3]. The major pathogenic mechanisms for HF development include haemodynamic overload, ischaemia, ventricular remodelling, and abnormal myocyte calcium cycling [4]. HF is classified on the basis of pathophysiological, anatomical, and functional characteristics. According to the left ventricular ejection fraction (LVEF), it is traditionally classified as HF with reduced ejection fraction (HFrEF) (LVEF < 40%) and HF with preserved ejection fraction (HFpEF) (LVEF $$\ge$$ 50%) [2, 5]. The predominant cause of HFrEF is ischaemic heart disease (IHD) [3, 6] whereas HFpEF has a less clearly defined aetiology with contribution from numerous risk factors including advanced age, hypertension, insulin resistance, and obesity [3]. Clinically, HF is a major global health problem that impacts primarily on the elderly, with a prevalence of 10% among those aged 65 and older [7]. This issue is compounded by ageing populations, with prevalence of the disorder predicted to rise substantially in the coming decades [8, 9]. The estimated 5-year HF mortality rate of 45% [10–12] is attributed largely to sudden cardiac death (> 50%) or to multiple organ failure as a result of widespread cardiac hypoperfusion [2, 6]. The progressive nature of HF also comes with a major economic burden, with an annual global cost of treatment being > $108 billion [13]. As such, it is paramount that individuals at increased risk or in the early stages of HF are identified to facilitate early intervention.

Current prediction of the development of HF is challenging for physicians because of its insidious onset [14]. Several risk prediction models have been developed but lack external validity [15, 16]. The emergence of cardiac biomarkers as potential clinical tools has been a topical research area over the past decade. The identification of an effective biomarker for HF would not only improve diagnosis but may also provide prognostic value and assist in the identification of high-risk patients that are likely to benefit from intensive therapy [17].

Brain natriuretic peptide (BNP) and N-terminal pro-BNP have been extensively studied and are currently utilised in clinical practice for the diagnosis of HF [18]. However, naturetic peptide levels are already elevated in the elderly and in those with anaemia, renal failure, and other cardiac conditions including acute coronary syndrome and myocarditis [18, 19]. Therefore, to improve diagnostic accuracy and risk stratification, a multi-biomarker approach has been proposed [17]. Recent studies have suggested fibroblast growth factor 21 (FGF21) as a promising biomarker candidate [20–22].

FGF21 exhibits a wide range of metabolic functions and has been described as a ‘cardiomyokine’, a protein with autocrine, paracrine, and/or endocrine actions that is essential for maintaining cardiac function [21, 23]. FGF21 protects against oxidative stress [24], hypertrophy and cardiac inflammation in animal, and in vitro cell culture studies [25]. However, in clinical studies, FGF21 levels are often elevated in cardiovascular diseases, including HF [21, 26, 27]. This review will discuss the potential role of FGF21 in HF pathophysiology and the basis for its use as a HF biomarker. The literature on the association between FGF21 and HF will be appraised critically along with preclinical evidence and the postulated underlying mechanisms.

## Heart failure

HFrEF is associated with impaired left ventricular contractility and weakening of the ventricular wall which is characterised by an eccentric remodelling pattern. This leads to chamber dilatation and a volume overload state and is related primarily with forward HF [6]. HFrEF is commonly a consequence of underlying coronary artery disease (CAD), valvular heart disease, and/or cardiomyopathies [6, 28]. Treatment of HFrEF involves modulation of the renin–angiotensin–aldosterone and sympathetic nervous systems. Current guidelines recommend all patients with HFrEF be treated with a combination of an angiotensin-converting enzyme inhibitor or angiotensin receptor-neprilysin inhibitor, plus a β-blocker, mineralocorticoid receptor antagonist, and most recently a sodium-glucose co-transporter 2 (SGLT2) inhibitor (regardless of diabetes status) [3].

HFpEF is caused by impaired left ventricular filling in which concentric ventricular wall hypertrophy leads to stiffening of the chamber wall and poor compliance [2]. This results in a pathological pressure overload state and backward HF that induces congestive sequelae such as pulmonary oedema. HFpEF is commonly found in older, obese, and female patients and is associated with atrial fibrillation (AF) and chronic hypertension [2, 6, 29]. Treatment options for HFpEF are limited and largely focus on symptomatic management, such as fluid control with diuretics [3]. Sodium-glucose cotransporter 2 inhibitors have been shown to reduce HFpEF hospitalisations but a robust reduction in mortality is yet to be demonstrated [30].

Currently, there is no serum biomarker that can accurately distinguish between HFpEF and HFrEF [31]. Treatment and prognosis are substantially different between the two subtypes and diagnosis of HFpEF is complex and currently based on echocardiogram findings and catheterisation. Thus, an effective biomarker would assist in streamlining diagnosis and improving early treatment decisions.

Beyond the naturetic peptides, various biomarkers have been proposed for HF diagnosis and prognosis across the HF spectrum [18]. These biomarkers represent different pathophysiological processes in HF such as inflammation, oxidative stress, chamber dilatation, and myocardial injury [18, 32]. Circulating inflammatory biomarkers for HF include interleukin-6, C-reactive protein, tumour necrosis factor-α, and galacetin 3. However due to inconsistent findings in the literature, these inflammatory biomarkers are not recommended for use in the clinical setting [33]. This is also the case with other biomarkers such as troponin, a marker of myocardial injury, which lacks specificity given that many conditions can cause myocardial stress [18]. As such, naturetic peptides are the only biomarkers that have sufficient evidence for use as HF biomarkers in clinical practice. With a high sensitivity, but a low specificity, measurement of a normal BNP level can rapidly exclude HF diagnosis, but an elevated level can only be interpreted in conjunction with conventional diagnostic techniques such as echocardiography [34]. Further, BNP does have the limitation of spuriously low levels in obese patients with HF [35]. Therefore, there is a need to identify better biomarkers for HF. These could be included in a multi-marker model or used individually. FGF21 has substantial promise as a novel biomarker as its circulating level gives an insight into several pathophysiological processes, such as oxidative stress, cardiac hypertrophy, and inflammation, that are involved in HF [24, 25].

## Metabolic functions of FGF21

The fibroblast growth factor family encompasses 22 factors involved in cell differentiation, cell proliferation, and embryonic development [36]. Fibroblast growth factors (FGFs) exert their effects by binding to one of four plasma membrane tyrosine kinase fibroblast growth factor receptors (FGFRs) [37]. FGFs are divided into seven subfamilies of which FGF19, FGF21, and FGF23 are members of the hormonal FGF subfamily [38]. With the exception of hormonal FGFs, all other members of the FGF family have a heparin binding domain that binds to heparin sulphate proteoglycans and initiates a FGFR-ligand interaction that activates downstream signalling cascades including the Ras/mitogen activated protein kinase and protein kinase C [38]. Their high affinity to heparin sulphate proteoglycans cause them to exert their effects in a paracrine function [39]. These paracrine FGFs can promote angiogenesis, cytoprotection, and tissue repair and are overexpressed in cancer [39].

Hormonal FGFs bind with low affinity to FGFRs and therefore require an obligate coreceptor, β-Klotho, for effective binding [38, 40, 41]. Whilst FGFRs are expressed in multiple cell types, expression of β-Klotho is tissue-specific and is found predominantly in the liver and adipose tissue [41]. FGF21 can act in an endocrine, paracrine, and autocrine manner and exhibits a diverse range of metabolic functions [37].

The metabolic function of FGF21 was first described in 2005 in a seminal publication that outlined its capacity to increase glucose uptake in adipocytes, and its ability to protect against obesity, hyperglycaemia, and hypertriglyceridemia in mice [42]. Importantly, no mitogenicity, hypoglycaemia, or weight gain was induced in healthy or diabetic mice by FGF21 administration at any dose or in transgenic mice with FGF21 overexpression [42]. Owing to this, the pathophysiology and potential pharmacological role of FGF21 in metabolic disease have been studied extensively [43].

**Fig. 1:**
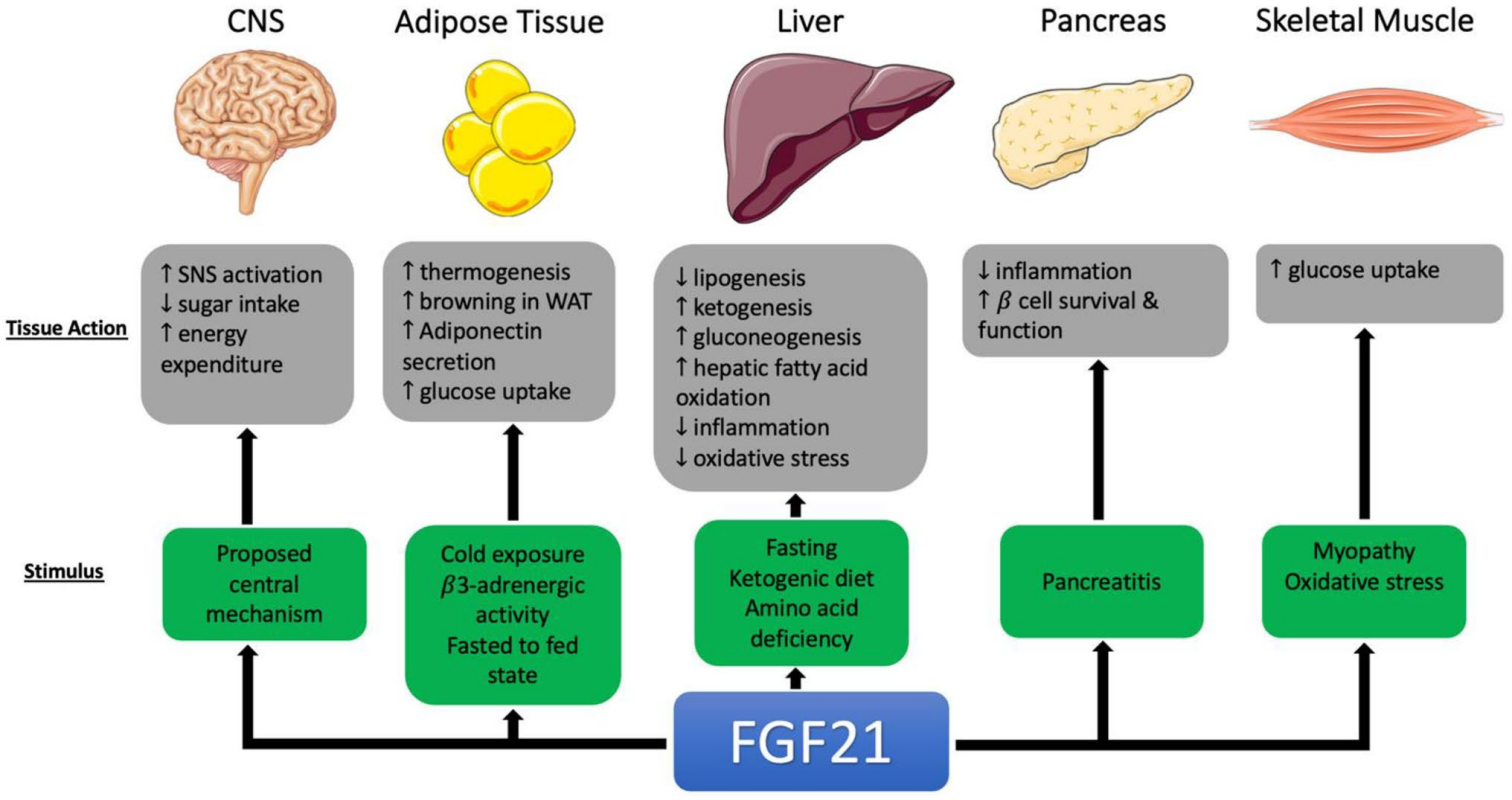
Overview of tissue specific actions of FGF21 in animal studies [38, 44, 45, 47–50, 52–54, 56, 59–61, 114]

FGF21 is expressed mainly in the liver, pancreas, skeletal muscle, white adipose tissue (WAT), and brown adipose tissue (BAT) [44]; however, under normal metabolic conditions, its circulating levels appear to be predominantly liver-derived [45, 46]. The tissue-specific actions of FGF21 are summarised in Fig. 1. In the liver, FGF21 is induced following extended fasting [45] which stimulates ketogenesis [47], gluconeogenesis [48], and hepatic fatty acid oxidation [49] and reduces lipogenesis [45]. FGF21 is also stimulated by ketogenic and low amino acid diets in mice, but not by ketogenic diets in humans [49–51].

FGF21 has thermogenic properties and thus contributes to energy expenditure through its action on WAT and BAT [52]. FGF21 facilitates the ‘browning’ of WAT, which upregulates thermogenesis [53]. FGF21 also induces the secretion of adiponectin, a hormone involved in fatty acid and glucose homeostasis, in WAT [54], which lowers blood glucose levels and increases insulin sensitivity. These effects are supported by studies showing that adiponectin knockout mice are resistant to the beneficial effects of FGF21 that alleviate insulin resistance, hyperglycaemia, and hypertriglyceridemia [54, 55]. FGF21 is also expressed in the pancreas [45] where it improves β-cell function and survival in rodent models of diabetes through the activation of the extracellular mitogen activated protein kinase 1 and 2 (ERK1/2) and Akt signalling pathways [56].

In skeletal muscle, FGF21 is synthesised under conditions of stress such as mitochondrial myopathies [38]. Studies have demonstrated its ability to increase insulin sensitivity by increasing glucose uptake in primary human skeletal muscle cells and isolated mouse skeletal muscle [57, 58] although there is no evidence that this also occurs in vivo [38].

A centrally acting mechanism for FGF21 that extends beyond peripheral organs has also been proposed. FGF21 is present in cerebrospinal fluid, where its level correlates positively with serum concentrations [59]. Intracerebroventricular injection of FGF21 also increases sympathetic activity, insulin sensitivity, and energy expenditure in rat models of obesity [60]. In obese mice with CNS-specific deficiency of β-Klotho, the beneficial effects of FGF21 on insulin sensitivity and body weight, as well as metabolic activity and gene expression in the liver, WAT, and BAT, were lost [61].

In humans, circulating FGF21 levels are elevated in metabolic syndrome, obesity, CVDs, diabetes, non-alcoholic fatty liver disease, mitochondrial myopathies, and cold exposure [26, 38, 62–69]. Given the beneficial metabolic effects of FGF21 that have been demonstrated in vitro, the paradoxical increase reported in these conditions is thought to be due to an FGF21 resistant state, caused by the impaired FGF21 signalling which results in the need for a higher FGF21 level to exert its beneficial metabolic effects [43, 70, 71]. This may explain the need for supraphysiological doses of FGF21 to achieve therapeutic efficacy in human clinical trial studies [72, 73]. Several animal studies have shed the light on the mechanistic basis of these observations by showing that FGF21 resistance is a consequence of reduced expression of β-Klotho and FGFRs in target tissues [70, 74], impaired FGF21 receptor interaction, and mitigation of downstream signalling pathways [70, 75]. In particular, the ERK1/2 pathway, considered the primary pathway for FGF21 intracellular signalling, is attenuated in diet-induced obesity mice, as evidenced by reduced expression of immediate early genes in liver and adipose tissue as compared to lean control mice [70].

## Pathophysiological role of FGF21 in HF: preclinical evidence

**Fig. 2:**
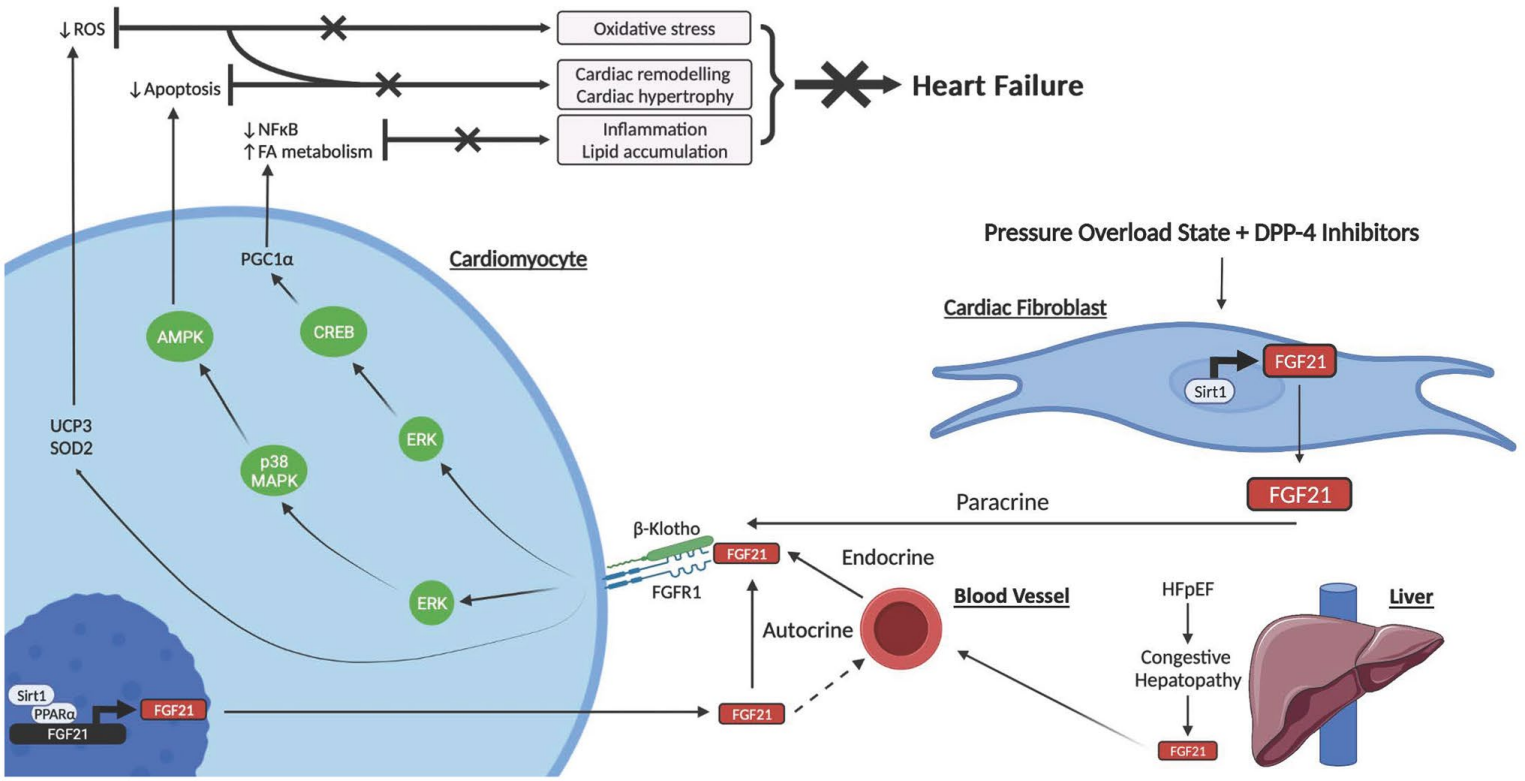
Schematic diagram of postulated molecular mechanisms for FGF21 cardioprotection against HF development, outlining FGF21 expression, and its endocrine, autocrine, and paracrine action in cardiomyocytes and cardiac fibroblasts [21, 24, 87]. Activation of the FGFR1/β-Klotho complex by FGF21 in cardiomyocytes stimulates the ERK pathway and phosphorylation of CREB protein, which increases PGC1 $\alpha$ levels. PGC1a downregulates the NFkB pathway and upregulates FA metabolism which collectively attenuate cardiac inflammation and lipid accumulation. FGF21 additionally upregulates UPC3 and SOD2 and activates the ERK mitogen activated protein kinase (p38 MAPK)/AMPK pathway. UPC3 and SOD2 reduce ROS and thus oxidative stress, and AMPK decreases apoptosis. A decrease in apoptosis and oxidative stress is associated with attenuation of cardiac remodelling and cardiac hypertrophy. Collectively, these mechanisms protect against HF development. FGF21 is additionally produced in cardiomyocytes in response to cardiac stress via the SIRT1-PPAR-α pathway which may act in an autocrine manner and stimulate the surface FGFR1/β-Klotho complex or enter the blood stream and contribute to alterations in energy metabolism in extracardiac organs. In a pressure overload state combined with administration of DPP-4 inhibitors, cardiac fibroblasts express FGF21 via SIRT1 which may contribute to cardioprotection via a paracrine interaction with cardiomyocytes. In response to congestive hepatopathy in HFpEF, the liver likely expresses FGF21 which feeds back onto the heart as a compensatory protective mechanism. Abbreviations: FGF21, fibroblast growth factor 21; Sirt1, sirtulin 1; PPAR α, peroxisome proliferator activated receptor α; FGFR1, fibroblast growth factor receptor 1; ERK, extracellular signal regulated kinase; CREB, cAMP responsive element binding; PGC1a, peroxisome proliferator-activated receptor γ coactivator 1-α; ROS, reactive oxygen species; NF-$\kappa$ B, nuclear factor $\kappa$ B; p38 MAPK, mitogen activated protein kinase; AMPK, AMP-activated protein kinase; FA, fatty acid; DPP-4, dipeptidyl peptidase-4; UCP3, uncoupling protein 3; SOD2, superoxidase dismutase 2

FGF21 was not initially thought to be related to the heart due to the modest expression of β-Klotho [76]. Despite this, its cardiac effects were first described by Planavila et al. in 2013 where FGF21 knockout mice exhibited increased signs of cardiac dysfunction with eccentric hypertrophy and induction of pro-inflammatory pathways [25]. In the same study, treatment with FGF21 reversed these effects in vivo as well as in cultured cardiomyocytes. This paper also established that endogenous production of FGF21 occurs in the heart in response to cardiac stress via the sirtuin 1 (SIRT1)-peroxisome proliferator–activated receptor α (PPAR-α) pathway [25], thus identifying the heart as both a target and source of FGF21. Following this study, FGF21 has been shown to be involved in various pathological processes which contribute to HF development such as oxidative stress, apoptosis and cardiac inflammation and lipid accumulation. The molecular mechanisms for the cardiac effects of FGF21 in relation to HF development are illustrated in Fig. 2.

### Oxidative stress

Induction of oxidative stress by excess production of reactive oxygen species (ROS) can cause structural damage and impair myocardial contractility, predisposing to HF [77–79]. In a further study by Planavila et al. (2015), FGF21, in an autocrine manner, was shown to upregulate genes such as uncoupling protein 3 (UCP3) and superoxide dismutase 2 (SOD2) in cardiomyocytes, which reduced oxidative stress [24].

### Apoptosis and cardiac remodelling

FGF21 also has a protective role in myocardial infarction survivors by inhibiting cardiomyocyte apoptosis [80] and mitigating myocardial remodelling and infarct size via an adiponectin-dependent mechanism [81]. Given that myocardial infarction is a common cause of HFrEF, FGF21 may reduce the risk of subsequent HF via this protective mechanism. Further, in a streptozotocin-induced diabetic mouse model, FGF21 upregulated the ERK1/2 mitogen–activated protein kinase (p38 MAPK)/AMP-activated protein kinase (AMPK) pathway to protect against lipotoxicity-mediated cardiac apoptosis in diabetes [82].

### Inflammation and lipid accumulation

There is mounting evidence supporting the involvement of cardiac inflammation and lipid accumulation in the development of HF [83–85]. The mechanistic basis of FGF21 cardioprotection regarding lipid accumulation and inflammation remains largely unknown. There is, nevertheless, some evidence to suggest that several signalling pathways are involved. For example, activation of the FGFR1/β-Klotho complex by FGF21 in cardiomyocytes stimulates the ERK1/2 pathway [21] and phosphorylation of cyclic AMP-responsive element-binding (CREB) protein, which increases PPAR-γ coactivator 1-α (PGC1 $\alpha )$ levels [25]. PGC1 $\alpha$ is a transcriptional coactivator involved in energy metabolism and oxidative stress [86]. Importantly, PGC1 $\alpha$ downregulates nuclear factor $\kappa$ B (NF-$\kappa$ B) pro-inflammatory pathways and enhances fatty acid oxidation, suggesting that the cardioprotective effects of FGF21 may, at least in part, be mediated by PGC1 $\alpha$.

### FGF21 in the cardiac fibroblast

Mouse cardiac fibroblasts express FGF21 under a pressure overload state, and treatment with the glucose-lowering medication, dipeptidyl peptidase-4 inhibitors, can further stimulate FGF21 expression in a dose-dependent manner [87]. This suggests that FGF21 expression in cardiac fibroblasts may contribute to cardioprotection through a paracrine interaction with cardiomyocytes. However, it should be noted this is the only study to report cardiac fibroblast expression of FGF21. Moreover, dipeptidyl peptidase-4 inhibitors have been found to be associated with an increased risk of HF events in human clinical trials, likely through the activation of the sympathetic nervous system [88]. Further research is therefore warranted to elucidate the role of FGF21 expression in cardiac fibroblasts in the pathophysiology of HF.

### Indirect cardioprotective effects of FGF21

FGF21 may also contribute to cardio-protection and attenuate HF development indirectly by decreasing blood pressure [89] and improving lipid profiles [90] as well as glucose and insulin homeostasis [42]. FGF21 may also mediate the effects of SGLT2 inhibitors on body weight reduction and lipolysis in adipose tissue and contribute, at least in part, to the reduction in all-cause and cardiovascular mortality in HF patients [91, 92]. In humans, an elevated FGF21 level in HF patients may be indicative of a FGF21-resistant state in response to chronic cardiac stress, as has been proposed in obesity [70], or a compensatory response to comorbid metabolic conditions, such as diabetes, which can precipitate HF [21]. Indirect evidence for FGF21 resistance and a reduction in its cardioprotective effects in the heart has been obtained by showing that the expression of FGF21 co-receptor, β-Klotho, is reduced in cardiac tissue of obese rats [93].

Future research should be directed at further elucidating the molecular mechanisms and pathways involved in FGF21 and HF development, particularly in the setting of HFrEF and HFpEF given their different pathophysiologies.

## Circulating FGF21 levels and atherosclerotic CVDs in humans

Several studies have assessed the association of FGF21 with CAD [94–98], subclinical atherosclerosis [99–101], and AF [102–104]. Given that CAD is a major risk factor for HF, it is important to understand these relationships. The consensus from two large meta-analyses is that CAD patients have elevated circulating FGF21 levels and that this is associated with poor prognosis [26, 105]. Lakhani et al. analysed FGF21 in different cardiometabolic disorders, including metabolic syndrome, diabetes, diabetic nephropathy, CAD, and cardiovascular motality, and reported elevated FGF21 levels were significant predictors of these disorders [105]. Although these findings were subsequently confirmed by Zhang et al. [26], both of these reports identified moderate-to-significant heterogeneity between studies which may impact the validity of the results.

On the other hand, results for subclinical atherosclerosis and AF are less conclusive. Ong et al. reported circulating FGF21 was not cross-sectionally related with subclinical atherosclerosis and did not predict cardiovascular events in apparently healthy people [99]. In contrast, another study found an association between carotid atherosclerosis and FGF21 independent of established CVD risk factors in women, but not in men [101]. Two further studies reported elevated circulating FGF21 levels in AF patients [102, 104], with a positive correlation of FGF21 levels with disease severity, whilst Hui et al. found no association between baseline FGF21 levels and incident AF in a cohort free of clinically apparent CVD [103].

## Circulating FGF21 levels and HF in humans

Chou et al. (2016) were the first to report a significant association of FGF21 levels with HF, focusing specifically on HFpEF. Using a cross-sectional study design [27], these investigators established that FGF21 provided prognostic value with elevated levels associated with increased mortality and HF readmission rates at a 1-year follow-up. However, this study was limited by a small sample size of 238 participants, and the control and HFpEF groups were poorly matched for age and sex [27]. This is an important confounder given patients with HFpEF were significantly older than the controls and that FGF21 levels increase with age [106].

An association between circulating FGF21 levels and HFpEF was confirmed by Ianos et al. who evaluated the diagnostic potential of several HFpEF biomarkers in a cohort of type 2 diabetic patients and identified FGF21 as the most promising biomarker [22]. In this study, FGF21 levels were significantly higher in diabetic patients with HFpEF, compared to diabetic patients without HF (mean 299.0 vs 146.8 pg/mL), and the association remained statistically significant after adjusting for clinical variables such as age, gender, and body mass index. Furthermore, at an optimal cut-off value of 217.40 pg/mL, FGF21 demonstrated high sensitivity (85%) and specificity (79.3%) for HFpEF [22]. Given that all of the participants of this study had confirmed type 2 diabetes, its generalisability is limited, and further studies in larger, more diverse populations are warranted.

Three studies have investigated FGF21 levels in HFrEF with the first published in 2019 by Holm et al. [107]. In this study FGF21 levels were evaluated in three groups: HFrEF patients with cardiac cachexia, HFrEF patients without cardiac cachexia and IHD patients with preserved ejection fraction [107]. Cardiac cachexia, a serious sequela of HF, is associated with unintentional weight loss and characterised by chronic inflammation and high mortality [6]. Patients with IHD were used as the control group given that IHD is a common comorbidity of HF with a metabolic risk profile similar to that of HFrEF. In contrast to the previous study [27], all groups were matched by age, renal function and sex. Plasma FGF21 levels were elevated in HFrEF patients with cardiac cachexia compared to those without cardiac cachexia and IHD patients. However, the association between FGF21 levels and cardiac function was not statistically significant, despite higher FGF21 levels being independently associated with higher interleukin-6 levels, lower muscle mass, higher total cholesterol, and lower HbA1c [107]. These observations suggest that the increased FGF21 levels in HFrEF patients with cardiac cachexia may be mediated by inflammatory and metabolic processes, rather than impaired cardiac function. However, the sample size in this study is small (*n* = 57), and the conclusions are limited because of its cross-sectional design.

In another small cross-sectional study, FGF21 levels were reported to be elevated in HFrEF patients compared to non-HFrEF controls, and an independent association of FGF21 levels with the combined endpoint of mortality and HF hospitalisation of HFrEF patients was reported [108]. These groups were well matched in terms of age and sex, and the prognostic value of FGF21 was high, with a sensitivity and specificity of 90% and 91%, respectively, at an optimal cut-off of 231.38 pg/mL [108]. These values were below those of BNP, with which FGF21 was compared in the study. This indicates that FGF21 likely cannot be substituted for BNP in HF diagnosis, although it may provide additional predictive power. Notably, all HFrEF patients in this study were classified as New York Heart Association (NYHA) functional status III or IV [108] and had very severe HF. Furthermore, Sommakia et al. also reported that FGF21 levels are elevated in HFrEF patients compared to healthy subjects [109]. Like the previous study, all the patients had end-stage HFrEF and were selected at the time of left ventricular assist device implantation so that cardiac tissue samples could be obtained. Analysis of tissue samples confirmed the presence of FGF21 protein in cardiomyocytes, although its gene expression was minimal in both failing and nonfailing hearts [109]. As circulating FGF21 levels are predominantly liver-derived [46], the liver is likely the principal extracardiac source of FGF21, and the authors proposed a hepatic to cardiac FGF21 signalling model in end-stage human HF. Circulating FGF21 levels are also associated with raised bilirubin levels but not elevated liver function enzymes, a pattern that is consistent with congestive hepatopathy and may be a signal for hepatic FGF21 production that feeds back to the heart where it exerts its cardioprotective functions [109]. Importantly, given congestive hepatopathy occurs in HFpEF, raised FGF21 levels in HFpEF may be a reflection of this compensatory protective feedback loop. This study may additionally support the hypothesis that HFrEF is, in part, a metabolic disease with alterations in fuel signalling proteins such as FGF21 from extracardiac organs such as the liver engendering changes in cardiac energy metabolism. Further research is needed to elucidate energy metabolism in HFrEF and how hepatic FGF21 production is increased in response to cardiac stress.

Finally, a further study by Gu et al. identified FGF21 as an independent predictor for poor prognosis in patients with dilated cardiomyopathy [20]. Furthermore, FGF21 levels were positively correlated with NYHA HF classification and negatively correlated with left ventricular ejection fraction in this study. FGF21 levels were also significantly elevated in the dilated cardiomyopathy patients compared to controls who were well matched with age, sex, BMI, and history of diabetes even though the incidence of AF was greater in dilated cardiomyopathy patients [20]. Despite this, the association of FGF21 levels with poor prognosis in dilated cardiomyopathy patients remained significant after adjusting for AF.

Indeed, raised FGF21 levels in HF in humans may appear deleterious given it is paradoxical to the cardioprotective functions exhibited in preclinical studies. Elevated levels, as proposed in obesity and evidenced by multiple animal studies, are likely, however, due to aberrant FGF21 signalling and an FGF21 resistant state [70, 75]. Additionally, as previously mentioned, elevated FGF21 levels may be a consequence of comorbid illnesses such as obesity and diabetes which can precipitate HF development [21].

Table 1 summarises the available clinical data on the association of FGF21 levels with HF. Overall, most of the studies in this area are limited by their small sample size, their cross-sectional design, their lack of ethnic diversity, and by not including both HFpEF and HFrEF patients (Table 1). No longitudinal studies on the relationship of circulating FGF21 levels with incident HF have been undertaken. Other limitations include differing baseline clinical characteristics of the study subjects such as type 2 diabetes and cardiac cachexia, as well as inconsistencies in adjustment models. Indeed, FGF21 is elevated in obesity, chronic kidney disease, metabolic syndrome, liver disease, and type 2 diabetes, and these pre-existing conditions may confound the findings. Additionally, there is minimal data on liver disease in all of these studies (Table 1). This is particularly important given that HF and liver disease often co-exist due to cardio-hepatic interactions [110]. As such, a higher prevalence of liver disease among HF groups could lead to false positive findings. Furthermore, there is a lack of data on ethnicity in most cohorts (Table 1), and given that some ethnic groups have an increased CVD burden [111], this is also likely to impact on comparison of study outcomes.

**Table 1:** Clinical evidence on the association of circulating FGF21 levels with HF

Reference	Population	Key findings	Limitations
**HFpEF**			
Chou et al. [27]	*n* = 238 •95 HFpEF patients •143 controls Patients followed up for 1 year to assess prognostic value of FGF21	•Elevated FGF21 levels were significantly associated with HFpEF •Higher baseline FGF21 levels predicted a higher mortality risk at 1 year	•Cross-sectional study design of FGF21 levels between HFpEF and controls which precludes causality •Small sample size •Control and HFpEF were poorly matched with traditional CVD risk factors more prevalent in the HFpEF group. In particular, HFpEF patients were significantly older •No data on pre-existing liver disease among patients •Single ethnic cohort (Chinese)
Ianos et al. [22]	*n* = 69 •40 HFpEF patients •29 controls Various CVDs were excluded	•Elevated FGF21 levels were associated with HFpEF	•Type 2 diabetic cohort •No data on pre-existing liver disease Single ethnic cohort (Caucasian)
**HFrEF**			
Sommakia et al. [109]	*n* = 60 •40 HFrEF patients •20 controls Patients with non-alcoholic fatty liver disease were excluded	•FGF21 levels were elevated in patients with HFrEF compared to controls •Tissue sample analysis revealed the presence of FGF21 in diseased cardiomyocytes, with elevated FGF21 levels likely originating from the liver	•Small sample size •Cross-sectional study design •Presence of comorbidities among HFrEF patients may confound the findings •HFrEF patients were end stage; thus, it is unclear whether findings can be generalised to patients with early-stage HF
Fan L, et al. [108]	*n* = 199 •128 subjects with HFrEF •71 controls Performed follow-up with a mean follow-up time of 13.36 months to assess 1-year mortality and HF readmission events In contrast to previous studies, patients with liver diseases were excluded	•Serum FGF21 was elevated in HFrEF •HFrEF patients with higher FGF21 levels had higher risk of 1-year mortality and heart failure readmission	•Small sample size •No multivariable regression analysis. Data was not adjusted for confounding factors •Single ethnic cohort (Chinese) •All HFrEF patients were NYHA functional class III or IV indicating a highly skewed population
Holm et al. [107]	*n* = 57 •19 patients with HFrEF and cardiac cachexia •19 patients with HFrEF and no cardiac cachexia •19 patients with IHD	•FGF21 levels were elevated in HFrEF and cardiac cachexia, as compared to the other two groups •Higher FGF21 levels were associated with higher interleukin 6 and lower muscle mass, but not cardiac function	•Cross-sectional study design •Small sample size •No data on pre-existing liver disease •Single ethnic cohort (Caucasian)
Gu et al. [20]	*n* = 321 •241 dilated cardiomyopathy patients •80 controls Patients followed up for a mean of 16 months. All-cause mortality and readmission were considered the end points Patients with liver disease were excluded	•Higher Serum FGF21 levels were independently associated with prognosis and severity of dilated cardiomyopathy •FGF21 levels were positively correlated with NYHA HF classification •FGF21 levels were inversely correlated with left ventricular ejection fraction in this study	•Cross-sectional study design assessing difference in FGF21 levels between groups •Patients with diabetes were not excluded •Single ethnic cohort (Chinese)

Research in larger, ethnically diverse, and clinically matched cohorts with adjustment for different confounding factors is thus important before FGF21 could be considered as a biomarker for HF. Such studies would provide insights into whether the relationship is linear and differs across ethnicity and HF subtypes. Additionally, there is a need to develop clinical cut-off values of FGF21 for HF diagnosis and prognosis and to investigate whether such cut-off values should be adjusted for different patients based on their HF medication regimens and clinical characteristics. Moreover, there is no study assessing the soluble forms of the FGF21 receptor complex components (such as soluble FGFR1) as potential HF biomarkers [112]. Since circulating FGF21 has a short half-life [113] and is elevated in HF, likely due to FGF21 resistance as a result of impaired FGF21 signalling, soluble forms of FGF21 receptor complex components could be a more stable HF biomarker than FGF21 itself.

## Conclusion

In summary, HF is a major global health problem, whose prevalence is expected to rise as populations age. FGF21 has cardioprotective effects in preclinical animal studies, and clinical evidence has demonstrated that it is elevated in both HFrEF and HFpEF. However, further research is warranted to evaluate whether circulating FGF21 can be used as a biomarker alone or as part of a multi-biomarker panel that includes BNP and other biomarkers to improve the accuracy of diagnosis and prognosis in individuals with HF. Based on the current evidence, the presence of numerous potential confounders and a lack of understanding of its precise role in HF pathophysiology means FGF21 may or may not be a biomarker of value in HF. FGF21 may be better suited to a multi-marker model alongside BNP to improve its low specificity. Larger longitudinal studies with greater statistical power are thus needed before the relationship of circulating FGF21 with incident HF can be evaluated. These studies would additionally provide insights into whether the relationship differs across subject characteristics including age, sex, and ethnicity. Such studies would enable thorough evaluation of FGF21 as a therapeutic target in HF. Importantly, the ability of FGF21 to distinguish between HFrEF and HFpEF would provide clinicians with improved early treatment decisions.

## Abbreviations

AF: Atrial fibrillation
BAT: Brown adipose tissue
BNP: Brain naturetic peptide
CAD: Coronary artery disease
CNS: Central nervous system
CVDs: Cardiovascular diseases
FGF21: Fibroblast growth factor 21
FGFRs: Fibroblast growth factor receptors
FGFs: Fibroblast growth factors
HF: Heart failure
HFpEF: Heart failure with preserved ejection fraction
HFrEF: Heart failure with reduced ejection fraction
IHD: Ischaemic heart disease
LVEF: Left ventricular ejection fraction
NYHA: New York Heart Association
ROS: Reactive oxygen species
SGLT2: Sodium-glucose co-transporter 2
WAT: White adipose tissue
